# Cystic echinococcosis: an emerging zoonosis in southern regions of Khyber Pakhtunkhwa, Pakistan

**DOI:** 10.1186/s12917-021-02830-z

**Published:** 2021-04-01

**Authors:** Shahid Niaz Khan, Rehman Ali, Sanaullah Khan, Sadia Norin, Muhammad Rooman, Noor Ul Akbar, Taj Ali Khan, Sumbal Haleem, Murad Ali Khan, Ijaz Ali

**Affiliations:** 1 Department of Zoology, Kohat University of Science and Technology Kohat-26000, Khyber Pakhtunkhwa, Pakistan; 2 Department of Zoology, University of Peshawar, Khyber Pakhtunkhwa, Pakistan; 3 Department of Zoology, Hazara University, Khyber Pakhtunkhwa, Pakistan; 4 Institute of Pathology and Diagnostic Medicine, Khyber Medical University, Peshawar, Pakistan; 5 College of Veterinary Sciences, Faculty of Animal Husbandry and Veterinary Sciences, University of Agriculture, Peshawar, Pakistan; 6 Department of Biosciences, COMSATS University, Islamabad, Pakistan

**Keywords:** Cystic echinococcosis, Khyber Pakhtunkhwa, Livestock, Hydatid cyst, Epidemiology, *Echinococcus granulosus*

## Abstract

**Background:**

Cystic echinococcosis (CE) is one of the principal causes of economic loss to the livestock industry because of its morbidity and mortality of food-producing animals and condemnation of important visceral organs. Pakistan being an agricultural country having an extensive livestock sector, is mostly practiced by poor people, which has a fundamental role in the economy. The present study was aimed to conduct a cross-sectional survey and PCR based confirmation of *Echinococcus granulosus* in sheep, goats, cows, and buffaloes from southern regions (three districts: Lakki Marwat, Bannu, and Karak) of Khyber Pakhtunkhwa, Pakistan. During the study, a total of 2833 animals were examined randomly including; sheep (*n* = 529), goats (*n* = 428), cows (*n* = 1693), and buffaloes (*n* = 183). Hydatid cysts were collected and examined for the presence of protoscoleces using microscopy. Detection of DNA was performed by using PCR and two mitochondrial genetic markers namely; *NAD-1* and *COX-1* were amplified.

**Results:**

The overall prevalence of CE was found to be (9%) among the examined animals. The hydatid cyst infection was highly prevalent in buffaloes (12%), followed by sheep (10%), cows (9%), and goats (5.1%). Cystic echinococcosis was more prevalent (10%; 96/992) in district Lakki Marwat followed by district Bannu (9%; 112/1246) and Karak (7%; 39/595). Female animals were more likely to be infected with CE (11.6%) than male animals (5.3%) (*p* = 0.001). Similarly, the infection was higher in the older group of animals as compared to younger (*p* = 0.001). Mostly (52.2%; *n* = 129) of hydatid cysts were found in the liver, while (64.4%; *n* = 159) cysts of the infected animals were infertile. PCR based identification confirmed the presence of *E. granulosus sensu stricto (s.s)* in the study area.

**Conclusion:**

Cystic echinococcosis was found to be highly prevalent in southern regions of Khyber Pakhtunkhwa and could be a potential threat to human health. Moreover, molecular sequencing and phylogenetic analyses should be carried out in future to identify the prevailing genotype (s) of *E. granulosus s.s*.

## Background

Cystic Echinococcosis (CE) is a serious zoonotic and pathologically important helminthic infection. It is caused by the hydatid cyst of *Echinococcus granulosus* [1] and mainly developed in the liver and lungs of intermediate hosts [2]. *E. granulosus* requires a definitive host (usually canines) and an intermediate host (herbivores/ occasionally humans) to complete its life cycle [3–5]. Cystic echinococcosis is highly endemic in herd keeping areas of the world [6] and cosmopolitan in distribution including South America, Eastern Europe, Russia, East Africa, Central Asia, China, Iran, Iraq, Syria, Jordan, Saudi Arabia, and Pakistan [7–9]. The disease has been included by the World Health Organization (WHO) in the list of neglected tropical diseases [5]. Hydatid cyst infection has a considerable impact both on human and animal health and causes important economic losses in livestock due to its morbidity and mortality in the endemic areas [10].

Cystic echinococcosis differs in nature within different geographic settings and host assemblages due to the morphological and biological variations among the *E. granulosus* population [11]. Mitochondrial and nuclear genetic markers are used to study molecular and genotypic polymorphism and several genotypes (G1-G10) have been recognized within *E. granulosus* [12]. The genotypes differ from each other based on host specificity, life-cycle and transmission patterns, developmental rates, pathogenicity, biochemistry, rate of human infectivity, and sensitivity to different drugs [13]. Prevalence studies and molecular identification play an important role in formulating control strategies and preventive measures which will in turn reduce the economic losses posed by CE [11].

Pakistan is an agriculture country and livestock has emerged as the largest subsector of agriculture over the years. More than 8 million rural families depend on agriculture and derive 35–40% of their income from livestock production [14]. The role of livestock in the gross domestic product (GDP) and economic sustainability is very crucial. Agriculture contribution to the national GDP is approximately 21% in which livestock shares about 11.9%. According to Pakistan Economic Survey (2019–2020), the population of sheep was estimated to be about 31.2 million, goat 78.2 million, cattle 49.6 million, and buffalo 41.2 million. The total meat production was reported to be almost 4, 708 tonnes [14]. Pakistan’s livestock sector has been considerably affected by CE and caused major economic losses [15, 16]. The economic loss to the livestock sector was reported to be 26.5 million (Pakistani Rupees) per annum due to parasitic assaults, while losses due to *E. granulosus* were assessed to be US$276.20 per 100 sheep and goats and US$165.72 per 100 cattle, camels, and buffaloes [17]. Regardless of the finest tropical varieties of livestock in Pakistan and well adaptation to the local conditions the output (milk, meat, and hide production) is not as good enough as it should be [18].

Studies regarding the prevalence of *E. granulosus* are few in Pakistan [8] and do not provide sufficient information on the geographical presence and aetiological agents of CE. Therefore, the current study has been designed to assess the presence of the parasite in livestock (cows, buffalos, goats, and sheep) and perform PCR based confirmation of *E. granulosus* in southern regions of Khyber Pakhtunkhwa. The present work will be essential to explore the prevalence of CE and may underpin research on the diagnosis, control, genetic diversity, and prevention of the disease in the future.

## Results

### Prevalence and risk factors

During the study, a total of 2833 (male = 1304; female = 1529) animals were examined and 247 (male = 69; female = 178) hydatid cysts were collected. The overall prevalence of CE was found to be (9%) among the examined animals. The disease was highly prevalent in buffaloes (12%), followed by sheep (10%), cows (9%), and goats (5.1%) (Table 1). This prevalence rate was statistically significant (*x*^2^ = 10.562; *p* = 0.014).

**Table 1:** Host and sex-wise prevalence of cystic echinococcosis

Host	Examined	Positive	Prevalence (%)	Pearson’s Chi-square (***x***^**2**^)	***p***-value
Buffaloes	183	22	12	10.562	0.014
Cows	1693	150	9		
Goats	428	22	5.1		
Sheep	529	53	10		
Total	2833	247	9		
**District**	**Sex**		**Total n/N (%)**		
	**Male n/N (%)**	**Female n/N (%)**			
Lakki Marwat	27/361 (7.5)	69/631 (10.9)	96/992 (9.7)	35.660	0.001
Karak	9/236 (3.8)	30/359 (8.4)	39/595 (6.6)		
Bannu	33/707 (4.6)	79/539 (14.6)	112/1246 (8.9)		
Total	69/1304 (5.3)	178/1529 (12)	247/2833 (9)		

^n^ Positive cases

^N^ Examined cases

^%^ Prevalence in percentage

Comparing the prevalence of CE in districts, it was found that CE was more prevalent (10%; 96/992) in district Lakki Marwat followed by district Bannu (9%; 112/1246) and Karak (7%; 39/595) (*x*^2^ = 4.761; *p* = 0.092). Sex-wise distribution revealed that female animals were more likely to be infected with CE (11.6%) than male (5.3%) (*x*^2^ = 35.660^a^; *p* = 0.001) (Table 1).

The age-wise prevalence of CE shown variations in animals of different age groups. Cystic echinococcosis was found to be highly prevalent in older age groups of animals than youngers. Animals of age less than 1 year were least susceptible to the parasite (1.3%), while animals of age group more than 5 years were highly susceptible (16%). This difference of CE infection in various age groups of animals was highly significant (*x*^2^ = 121.225; *p* = 0.001) (Table 2). The organ-wise prevalence of CE was also observed and 129 (52.2%) hydatid cysts were found in the liver, 98 (40%) in lungs, while only 20 (8.1%) in liver and lungs of the infected animals (*x*^2^ = 2833.0; *p* = 0.001) (Table 2).

**Table 2:** Age and organ-wise prevalence of cystic echinococcosis

Age (year)	Animal examined				Outcome			
	Buffaloes	Cows	Goats	Sheep	Positive	Prevalence (%)	Pearson’s Chi-square (***x***^**2**^)	***p***-value
< 1	64	530	196	211	13	1.3	121.225	0.001
1–5	72	723	146	202	124	11		
> 5	47	440	86	116	110	16		
**Organ**					**Total**			
Liver	14	80	09	26	129	52.2	2833.0	0.001
Lungs	05	55	12	26	98	40		
Liver + lungs	03	15	01	01	20	8.1		
Total					247	100		

Hydatid cysts after collection were categorized into 3 categories based on their fertility (fertile, infertile, calcified), viability (viable and non-viable), and morphology (unilocular and multilocular). On the basis of fertility, most of the cysts were infertile (64.4%; *n* = 159), followed by fertile (28.4%; *n* = 70), and calcified (7.3%; *n* = 18) (*p =* 0.25) (Table 3). While only (10.2%) of the cysts showed viability and the remaining (89.8%) were recorded to be non-viable cysts (*p =* 0.48). Moreover, among the collected cysts, it was also reported that (98%) were unilocular and (2%) were multilocular (*p =* 0.63) (Table 3).

**Table 3:** Types of cysts observed in the study

	**Animals examined**						
**Cyst type**	**Buffaloes**	**Cows**	**Goats**	**Sheep**	**Total**	**Prevalence (%)**	***p-*value**
**Cyst based on fertility**							
Fertile	02	48	07	13	70	28.4	0.25
Infertile	20	87	15	37	159	64.4	
Calcified	00	15	00	03	18	7.3	
Total					247	100	
**Cyst based on viability**							
Viable	00	20	00	05	25	10.1	0.48
Non-viable	22	130	22	48	222	90	
Total					247	100	
**Cyst based on morphology**							
Unilocular	22	145	22	53	242	98	0.63
Multilocular	00	05	00	00	05	2	
Total					247	100	

### PCR based confirmation

**Fig. 1:**
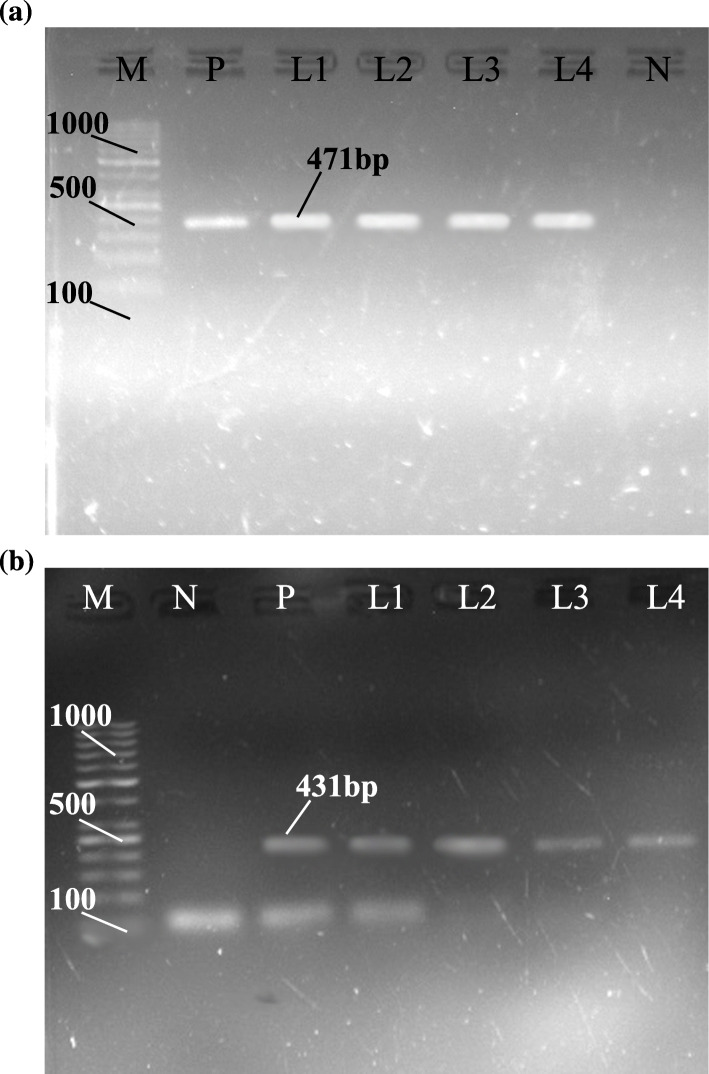
Gel electrophoresis of amplified (**a**) *NAD-1* and (**b**) *COX-1* genes. **M**=indicates DNA ladder; **P**=indicates positive control (*E. granulosus* G1); **N**=indicates negative control (water); **L1**-**L4**=indicate positive samples

DNA samples, from fertile cysts, were amplified by PCR using species-specific primers and generated fragments of *NAD-1* (471 bp) and *COX-1* (431 bp) genes for confirmation of *E. granulosus* (Fig. 1). PCR analysis confirmed the presence of *E. granulosus s.s* in the three districts.

## Discussion

Cystic echinococcosis is one of the principal causes of economic loss to the livestock industry in terms of causing morbidity and mortality of food-producing animals, and condemnation of important visceral organs. Hence, it is reasonable to find reliable information for monitoring the epidemiological status of CE to provide baseline data for future comparison, which will be of great importance in controlling and prevention CE.

In this cross-sectional survey, the overall prevalence of CE was reported to be 9.0% in the animals examined during this study. In Pakistan, the prevalence of CE has been previously reported by other investigators in different regions of the country. Ahmed et al., (2006) has reported 30.4% prevalence in Quetta [19], Khan et al., (2010) reported 6.43% in cattle in Lahore [20], Iqbal et al., (2012) reported 7.2% in Lahore [21], while Mustafa et al., (2015) reported 2.71% of CE prevalence in Punjab [22]. The results of the present study suggest that the hydatid infection is present in the region at a moderately endemic level. No rules and guidelines are followed concerning animal slaughtering, mostly animals are slaughtered without any supervision of the meat inspector(s) in the abattoirs, small towns, and other rural communities, where appropriate disposal of offal and infected organs is not practiced [23], which may contribute to the persistence of the disease. In contrast, the prevalence of CE in this study is very low than the prevalence described in other regions of the world: in Moldova 59.3% [24], Ethiopia 20.5% [25], and Libya 15% [26]. This difference in the prevalence might be due to the difference in sample size, geographic variations, and different climatic conditions.

In Pakistan, the incidence of CE has been described in different types of animals such as buffaloes, cows, goats, sheep, and camels. Ahmed et al., (2006) has observed the incidence rate of CE in goats (21.73%) and sheep (37.29%) in Quetta [19], Latif et al., (2010) has observed the prevalence in buffaloes (7.19%), cattle (5.18%), goats (5.48%), sheep (7.52%) and camel (17.29%) in Punjab [17], while Iqbal et al., (2012) has reported the prevalence rate of CE in goats (6.21%) and sheep (8.25%) in Lahore [21]. Similarly, Mustafa et al., (2015) has detected CE in cattle (2.44%), goats (2.44%), and sheep (3.24%) in Punjab province [22], and Haleem et al., (2018) has observed the presence of CE in buffaloes, cows, goats, and sheep 15.88, 15.79, 3.25, and 15.38%, respectively [16]. The results of the current study are in parity with the aforementioned studies as we described the incidence rate in buffaloes (12%), sheep (10%), cows (9%), and goats (5.1%). Our findings reveal that CE is more prevalent in buffaloes and sheep as compared to cows and goats. This result is in agreement with the earlier studies conducted in Greece, Iran, and India [4, 27, 28]. Haleem et al., 2018 also concluded that CE was highly prevalent in buffaloes, however, they are unlikely to spread the disease as mostly infertile cysts were described in buffaloes [16]. Similarly, the low prevalence of CE in goats may be attributed to the grazing style of these animals, as goats typically feed on leaves, upper parts of plants, and tall bushes, which make them less likely to contact with the infective stage of parasite (eggs) and thus a low risk of infection in goats than sheep and cattle [29].

There was a difference in the rate of prevalence of CE in different areas; the prevalence was high in the Lakki Marwat district as compared to Bannu and Karak. Similar trends of variation in the prevalence have been reported by other researchers [16, 24–26, 30], suggesting that CE prevalence varies from region to region or even in different regions/parts of the same country. Most probably this variation in the prevalence may be associated with geographical distribution, social and cultural activities of the local people, and a good sanitary system. Furthermore, slaughtering of animals at home without meat inspection, improper handling of byproducts and offal, dogs access to raw carcasses, and lack of health education in dog’s and animal’s owners [31, 32] may also be the contributing factors to CE prevalence in different areas.

Female animals were found to be more susceptible (12%) to the disease than male animals (5.3%). Other workers have also confirmed that the infection rate is higher in females as compared to males [16, 20, 21, 23, 28, 33–36]. The susceptibility driven gender-wise infection difference could be due to several reasons (i) female are kept for a longer period due to milk production and reproductive purposes than male [36], (ii) males are usually slaughtered at an early age [20], (iii) and variation in the management of livestock usually females are managed near houses for milking purposes, which expose them more to come in contact with infected dogs [16], consequently the likelihood of having high incidence in female than in the male. About the correlation of CE and age of the host, it was found that the disease was relatively high prevalent in animals of age > 5 years followed by 1–5 years and < 1 year. Similar observations were also reported by other researchers that usually animals with older age were more vulnerable to the parasite as compared to younger animals [4, 16, 33, 36–42]. The feasible reasons for a higher rate of infection in older animals may be attributed to several factors; hosts with older age have a long duration of exposure to the parasites, thus they are more likely to get infected, in older animals, the cysts gain reasonable sizes and become easy to diagnose, and aging is commonly associated with various diseases, hence, chronic nature of CE justifies its prevalence in old animals [36]. The tendency of CE in gender and age-wise shows that the infection is strictly associated with age rather than gender.

The organ-wise distribution of CE revealed that hydatid cysts were prevalent in the liver (52.2%) followed by the lungs (40. Several studies conducted in Pakistan [15–17, 19, 36] and other countries [4, 28, 43] reported similar findings. Though, CE development may also occur in other organs and tissues of the body when oncospheres reach the circulatory system [44]. For instance, some studies reported the involvement of other organs like the heart, kidney, and spleen, etc. [36, 43]. It might be due to the reason that the liver collects blood with the oncosphere through the bile duct after blood circulates from the duodenum, and if the oncosphere is not filtered in the liver, it might be passed to other organs like the lungs, heart, kidneys, and spleen, etc. [45].

Observations about the frequency of fertile and viable hydatid cysts from livestock provide vital indicators about the transmission of CE, as they act as the main source of infection to the definitive host by ingestion of fertile cysts. Cysts depending on geographical situation, kind of infected hosts, site, size, and type of cyst may have different fertility rates. The highest rate of cyst fertility was observed in the liver of cows, sheep, and goats. In cows and goats, the fertility rate was higher in the liver as compared to the lungs of the infected animals, whereas, in sheep, it was higher in the lungs than the liver. The findings of the present study are supported by those from other countries in which liver cysts were found to be more fertile than the lungs [21, 28, 46–48]. In contrast, some studies indicated that cysts found in the lungs were more fertile than liver [49–51]. This difference in the rate of fertility may be because different strains of *E. granulosus* are present in different areas and infection may occur as a result of a mixture of strains. No fertile cyst was reported in buffaloes in this study, these results are in agreement with those obtained in France, Italy, and Spain [41, 52, 53], indicating that, except for infection by their attributed genotype, they are not a major active intermediate host for the parasite but a dead-end host [54].

From the findings, it is evident that infertile and calcified cysts were also observed. Similar results of infertile cysts are also reported in other studies [43, 55, 56]. The infertility of hydatid cysts might be due to the inherent inability to reproduce, but in the majority of cases, it could be due to some abnormal local conditions. Besides, the availability of nourishment may be the most significant factor and influenced by the location of hydatid cyst and the condition of the adventitious coat. Sterile hydatid cysts may also be due to infection by unspecific strain [57]. Cyst viability assessment indicated that only a small number of the cysts were viable. Viable cysts were exclusively present in cows and sheep. The viability of cysts was also previously studied by other researchers [16, 28, 39, 58]. The viability of protoscolices may vary from host to host, it might be attributed to the difference in the immunological response of each host. During the study, it was observed that 2.0% of the collected cysts were multilocular, while the rest of the cysts were unilocular. This indicates that *E. granulosus* and *E. multilocularis* coexist in the study area, however, further epidemiological and molecular studies will be required to investigate *E. multilocularis* and confirm the existence of this parasite species.

To the best of the author’s knowledge, this is the first study of its kind to investigate the PCR based confirmation of *E. granulosus* in livestock of Khyber Pakhtunkhwa. In Pakistan, few studies reported the molecular epidemiology of *E. granulosus* in various intermediate hosts. The PCR based confirmation further emphasized that *E. granulosus* is prevailing in the southern regions of Khyber Pakhtunkhwa. These results are supported by earlier studies in Pakistan, which confirmed different genotypes of *E. granulosus* through molecular characterization and sequence analysis [15, 17, 36, 59, 60]. However, in the present study, sequencing was not performed for further confirmation of the genotypes which is the limitation of this study. There is a dire need and scope of research to investigate *E. granulosus* on the molecular level and further in-depth analyses are required in future research. Moreover, CE is an underrated zoonotic infection and needs immense attention from Govt. organizations and research institutions from all over Pakistan. Studies on prevalence, epidemiological status, risk factors and common practices leading to CE transmission, control, and molecular identification should be carried out to further explore this zoonotic infection. This study provides baseline information about CE, which could have a significant impact on the public health of Pakistan. It will help to explore the potential source of CE in humans and implement effective control programs.

## Conclusion

From the study, it is concluded that CE is prevalent in the southern regions of Khyber Pakhtunkhwa. Cystic echinococcosis was highly prevalent in female animals and animals of older age. The liver was found to be the most frequently infected organ. PCR based analysis revealed the presence of *E. granulosus s.s*, however, genetic characterization should be carried out in future to explore the circulating genotype/strain (s).

## Methods

### Study area

**Fig. 2:**
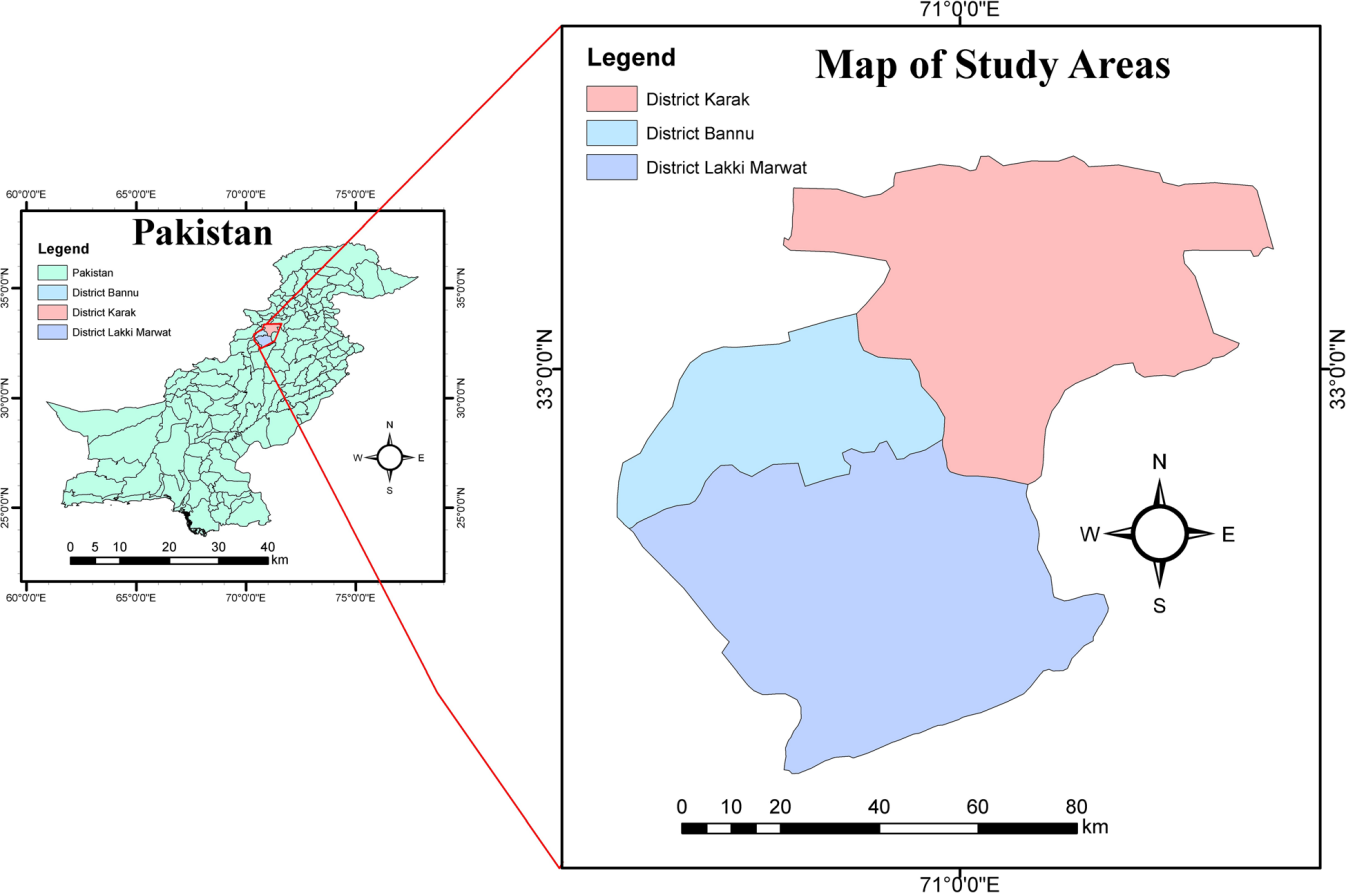
Map of the study area (Drawn using a software “ArcGIS)” (https://desktop.arcgis.com/en/)

The study was carried out in three districts, namely, Lakki Marwat, Karak, and Bannu of Khyber Pakhtunkhwa, Pakistan (Fig. 2). Agriculture and farming are the major sources of income in these districts and dogs cohabiting with domestic animals is a very common practice. However, most of the rural population has no awareness of zoonotic infections, stray dogs are common, people keep dogs as pets and for security purposes, grazing of animals is common, during Eid-ul-Azha (religious occasion) animals are slaughtered and their carcasses are not disposed of/buried properly. Due to zoonotic nature of the disease and keeping dogs near domestic animals make the situation ideal for the parasite to circulate and continue its life cycle by infecting domestic animals and humans as well. Lakki Marwat is situated between 32^∘^161 N latitudes and 70^∘^191E longitudes at an altitude of 200–1000 m above sea level. This district covers an area of 3164 km^2^ with a cultivated area of approximately 116,900 ha. The indigenous people of the district are Marwat tribes, but a small proportion of other tribes also settled here. Transport and minerals are the main sources of economy in the urban area, and agriculture is the primary livelihood of the rural population [61]. Karak region is situated in the south of Khyber Pakhtunkhwa with a total area of 600 km^2^ and lies between 70 and 40^∘^ to 71–30^∘^N latitude and 32–48^∘^ to 33–23^∘^E longitude. Bannu consists of a total area of 877 km^2^ with an urban population of 49,593. It lies within the Karakoram mountain range between 32^∘^43 to 33^∘^06 N latitude and 73^∘^20 to 70^∘^07E longitude. The total cultivated area is about 33,000 acres [62].

### Sampling and post-mortem examination

**Fig. 3:**
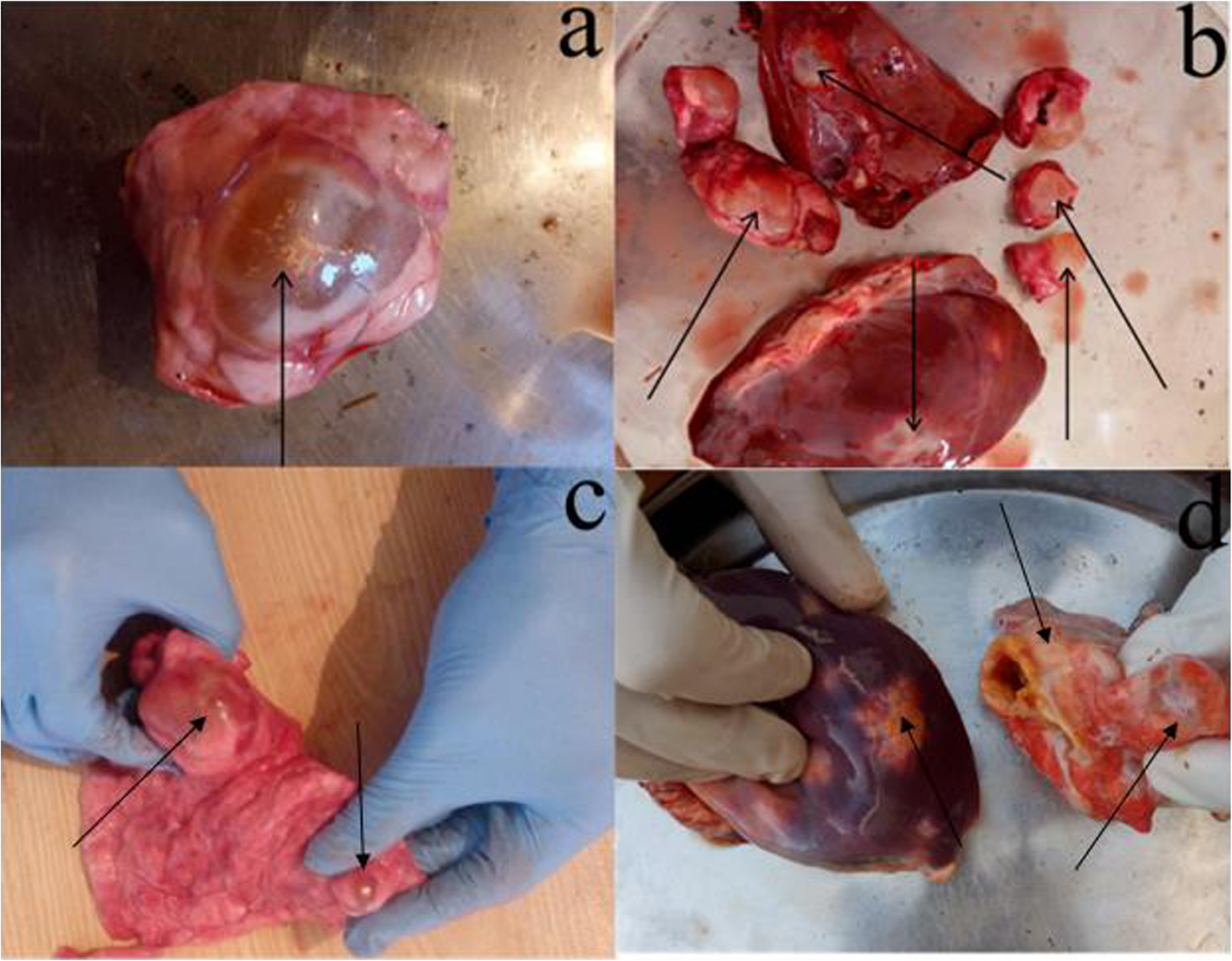
Cysts collected during this study (**a**, **c**) represents cyst of the lungs (**b**) represents cysts found in the liver (**d**) several small cysts in the liver (left) and calcified cyst in lungs (right)

Before the data collection, consent from the meat inspector (in the case of slaughterhouses) and head of the butchers’/slaughterers’ union (in case of butcher shops) was taken and a brief purpose of the study was explained. Different slaughterhouses and butcher shops of the above-mentioned three districts were visited multiple times a month for sampling. A randomized sample collection approach was employed to collect hydatid cyst (s). Post-mortem examination of a total 2833 animals were carried out including; buffaloes (*n* = 183), cows (*n* = 1693), goats (*n* = 428), and sheep (*n* = 529) from Jan-2018 to Dec-2018. The teeth of the animals were examined to estimate their age. The slaughtered animals were visually observed for hydatid cysts or palpated and organ (s) was sliced in case of any ambiguity. Hydatid cyst samples were collected under aseptic conditions from the liver and lungs of slaughtered animals (Fig. 3). The animal was defined as positive if one or more cysts were found and as negative if no cyst was found. The detailed history regarding the hydatid cyst infection was recorded on a prescribed proforma comprised of the district, type of animal, sex of the host, infected organ (liver, lungs, or both), and type of cyst (unilocular or multilocular). The cyst samples were transferred in cool boxes with sterile normal saline to the Molecular Parasitology and Virology Laboratory, Department of Zoology, KUST for further experimental analysis.

### Microscopical examination

The individual cyst was washed with phosphate-buffered saline (PBS) and fluid was aspirated from each cyst via a 5 mL syringe under aseptic conditions into sterile falcon tubes. The tubes were stored at -4 °C until further analysis. The cystic fluid was subjected to centrifugation for 8 min at 3000 rpm, the supernatant was discarded and the pellet was left at the bottom. The precipitate was shacked well and one drop of fluid was placed on a glass slide and covered with a cover-slip, then examined under a light microscope at 40X magnifying lens for the presence of protoscoleces by using 0.1% aqueous eosin stain. The presence of protoscoleces was an indicator of cyst fertility and vice versa [63]. The viability of cysts was further confirmed by observing the amoeboid like peristaltic movement/flame cells activity of protoscoleces [64].

### DNA extraction

Total genomic DNA was extracted from the protoscoleces of *E. granulosus* cyst fluid by Phenol-chloroform method according to the standard procedure of Wang et al., 2014 [2]. The brief procedure of DNA extraction was: the cystic fluid (2.5 mL) was taken in an Eppendorf tube and was centrifuged (Biobase China) for 10 min at 3000 rpm (rpm) at room temperature. The supernatant was discarded and the pellet was rinsed 3–4 times with sterile saline and repeatedly centrifuged at 3000 rpm for 10 min and washed with 70% ethanol. The protoscoleces (30 μL) were taken in a new tube and 300 μL of lysis buffer was added and mixed by vortex (Biobase, China) for 5 s. The suspension was placed on a heat block at 94 °C for 20 min and then allowed to cool down at room temperature. The proteinase K (30 μL) (Thermo Fisher Scientific, USA) and lysis buffer (300 μL; pH = 8) were added, vortexed, and placed in a water bath at 56 °C for 1 h. The suspension (300 μL) was taken in a new tube and phenol (300 μL) was added and centrifuged at 5000 rpm for 5 min. The supernatant (300 μL) was transferred to a new tube and chloroform (300 μL) was added and mixed before spinning at 5000 rpm for 5 min. The supernatant (300 μL) was again transferred into a new test tube and (300 μL) of isopropanol + 0.1 volume of sodium acetate was added and kept at -20 °C for 20 min. The test tube was subjected to centrifugation for 15 min at 14,000 rpm. The sediment was rinsed by adding 70% ethanol (300 μL) and centrifuged for 5 min at 5000 rpm. The resulting pellet was dissolved in (150 μL) of deionized water. The DNA was stored at -4 °C until further molecular analysis.

### Polymerase chain reaction (PCR) and gel electrophoresis

The genomic DNA was amplified through PCR by using forward JB11.5 (5′-TTATGGTAGATATTATAG-3′) and reverse JB12.5 (5′- CACACACATAAAACAAGC-3′) primers of *NAD-1* gene [65], while forward (5′-TTTTTTGGGCATCCTGAGGTTTAT-3′) and reverse (5′-TAAAGAAAGAACATAATGAAAATG-3′) primers of *COX-1* gene as described by [66]. Thermal cycler (Kyratec; Model-SC300G, Australia) was used to amplify the desired genes, keeping the PCR reaction volume of about 25μL containing PCR Master mix (SolisBiodyne, Estonia) 12.5μL (MgCl_2_, dNTPs, *Taq* DNA polymerase), forward and reverse primers (Macrogen, Korea) of 1.5 μL each, 5.5 μL PCR water (dH_2_O), and 4 μL of extracted DNA. The applied PCR conditions were: initial denaturation at 94 °C for 5 min; followed by 35 cycles of denaturation at 94 °C for 30 s, annealing at 48 °C and 52 °C (*NAD-1* and *COX-1*, respectively) for 45 s, while extension, and a final extension at 72 °C for 45 s (each). Water was used as a negative control and *E. granulosus* G1 genotype was used as a positive control provided by the Molecular Virology Lab of COMSATS University, Islamabad (CUI). After amplification, the PCR amplified products were stored at -4 °C for further analysis.

Agarose gel (2%) was prepared and the final PCR amplicons were separated via gel electrophoresis and visualized through the Gel Doc system (UVP BioDoc-It Imaging System).

### Data analysis

The epidemiological data of different variables were analyzed using the statistical tool IBM SPSS Statistics (Version 23). Chi-square Pearson’s test (*x*^2^) and One Way ANOVA were used for statistical analyses. The *p-*value (0.05) was considered to be statistically significant.

## Data Availability

The datasets used/or analysed during the current study are available from the corresponding author on reasonable request.

## Abbreviations

CE: Cystic echinococcosis
*COX-1*: *Cytochrome c oxidase 1*
*NAD-1*: *Nicotinamide adenine dinucleotide dehydrogenase subunit 1*
GDP: Gross domestic product
KUST: Kohat University of Science and Technology
DNA: Deoxyribonucleic acid
PCR: Polymerase chain reaction
SPSS: Statistical package for social sciences
